# Comparative evaluation of MALDI-ToF mass spectrometry and Sanger sequencing of the *16S*, *hsp65*, and *rpoB* genes for non tuberculous mycobacteria species identification

**DOI:** 10.3389/fcimb.2025.1612459

**Published:** 2025-07-28

**Authors:** Angel Sebastian Rodriguez-Pazmiño, Elsy Carvajal, Darwin Paredes-Núñez, Jose Echeverría, Joselyn Calderon, Solon Alberto Orlando, Henry Parra Vera, Miguel Angel Garcia-Bereguiain

**Affiliations:** ^1^ One Health Research Group, Universidad de Las Américas, Quito, Ecuador; ^2^ Instituto Nacional de Salud Pública e Investigación, Guayaquil, Ecuador; ^3^ Universidad Católica Santiago de Guayaquil, Guayaquil, Ecuador; ^4^ Universidad Ecotec, Guayaquil, Ecuador; ^5^ Universidad Espíritu Santo, Guayaquil, Ecuador; ^6^ Centro de Investigación Microbiológica, Guayaquil, Ecuador

**Keywords:** Sanger sequencing, MALDI-ToF MS, non-tuberculous mycobacteria, Ecuador, clinical performance

## Abstract

Non tuberculous mycobacteria (NTM) infections are increasing globally, underscoring the critical importance of accurate species-level identification for effective clinical management. This study aimed to evaluate the use of three conserved markers in the mycobacterial family (*16S*, *hsp65*, and *rpoB*) for NTM identification through Sanger sequencing, comparing the results to those obtained using MALDI-ToF MS. A total of 59 clinical NTM isolates from plastic surgery patients, previously characterized by MALDI-ToF MS, were analyzed. These isolates underwent DNA extraction, PCR amplification, and Sanger sequencing. Species identification was performed through phylogenetic analyses of each marker individually and concatenated as a multi locus sequencing approach. Concordance between MALDI-ToF MS and Sanger sequencing was assessed using Cohen’s Kappa statistical analysis. Cohen’s Kappa values indicated moderate concordance of 0.46 for *16S*, 0.51 for *hsp65*, and 0.69 for *rpoB*. Concatenated phylogenetic analysis yielded improved concordance values of 0.71 for (*16S* + *hsp65)*, 0.76 for (*16S* + *rpoB)*, 0.69 for (*rpoB* + *hsp65)*, and 0.72 for (16S + *hsp65* + *rpoB*). Our results show that NTM identification is more accurate when employing a multi locus sequencing approach. Notably, the combination of *16S + rpoB* outperformed the three-marker concatenation, offering the highest concordance for species-level identification. NTM identification is challenging, and concatenated phylogenetic analysis of two or more gene fragments should be used when MALDI-ToF MS or whole genome sequencing is not available.

## Introduction

Non tuberculous mycobacteria (NTM) include mycobacteria that are different from those that cause tuberculosis and leprosy. They are ubiquitous in the environment and mostly harmless to humans, but some NTM species can lead to either pulmonary or extrapulmonary infections of skin, soft tissue, lymphatic, and disseminated infections, and even nosocomial outbreaks when medical equipment is inadequately disinfected/sterilized (McGrath and Anderson, 2007a; Zhang et al., 2023). NTM infections are rising not only in immunocompromised patients becoming an emerging public health issue worldwide (McGrath and Anderson, 2007b). NTM pulmonary diseases account for the majority of NTM diseases, and it has been associated with several NTM species, including *M. avium* complex, *M. abscessus* complex, *M. kansasii*, *M. fortuitum*, *M. gordonae*, *M. xenopi*, *M. chelonae*, and *M. malmoense* (Griffith et al., 2012). NTM infections usually require difficult and long antibiotic treatments, and clinical guidance for management of NTM infections highlights the importance of identification at the species level to choose the appropriate antibiotic treatment (Griffith et al., 2012).

Matrix-Assisted Laser Desorption Ionization-Time of Flight Mass Spectrometry (MALDI-ToF MS) is used to identify microorganisms (including mycobacterial species) based on their unique spectral fingerprint from extracted proteins (Hrabák et al., 2013). For many years, identifying mycobacterial species using MALDI-ToF MS has been challenging, primarily because of their intricate cell wall structure and fastidious growth. Since its initial use for identifying mycobacteria, significant progress has been made in employing this technique to identify NTM species (Alcaide et al., 2018). Advancements in extraction methods have improved both the accessibility and quality of proteins for analysis using MALDI-ToF MS. In addition, the number of mycobacterial spectra, essential for the identification process in MALDI-ToF MS, has steadily increased over the years (Ravva et al., 2017; Ricchi et al., 2017; Rodriguez-Temporal et al., 2017; Alcaide et al., 2018). Despite advancements, identifying many mycobacterial species remains challenging through proteomic techniques that are not always accessible, especially in low- and middle-income countries (LMICs).

Sequencing-based methods, such as Sanger sequencing, have become useful techniques for NTM identification due to their level of resolution and flexibility (Tortoli, 2010; Kim et al., 2022). Phylogenetic and taxonomic studies of NTM species have traditionally relied on the analysis of the *16S rRNA* gene, due to its conserved nature and universal presence in bacteria. However, its limitations include high genetic similarity among species that complicate precise differentiation (Devulder et al., 2005). To address this, combining *16S rRNA* gene analysis with additional markers, such as *hsp65* and *rpoB*, has proven to be an effective approach for identifying mycobacterial species (Maleki et al., 2017). The *hsp65* gene, which encodes the 65 kDa heat shock protein, is highly conserved across NTM species but includes hyper variable regions that enhance its discriminatory power (Telenti et al., 1993). Similarly, the *rpoB* gene, which encodes the β-subunit of RNA polymerase, contains conserved sequence regions interspersed with highly variable regions, making it a valuable alternative or complementary tool for species identification (Boor et al., 1995; Adékambi et al., 2003). Other procedures, such as whole-genome sequencing (WGS), have also proven effective for identifying NTM (He et al., 2020; Dohál et al., 2021). However, due to the higher costs, processing levels, and requirement of trained personnel to work with WGS, its implementation in NTM diagnosis is limited compared to Sanger sequencing (Lorente-Leal et al., 2022).

Public health’s critical need to monitor NTM species underscores the importance of reliable diagnostic techniques for accurate species identification. This study aimed to carry out a methodological validation of Sanger sequencing and phylogeny analysis of the three widely used genetic markers *16S*, *hsp65*, and *rpoB* for NTM species identification using MALDI-ToF MS as a gold standard.

## Methodology

### Non tuberculous mycobacteria isolates

Fifty-nine NTM isolates were cultured using the Kudoh Ogawa method (Franco-Sotomayor et al., 2020). NTMs were isolated from skin purulent lesions caused by plastic surgery from a clinical laboratory in Guayaquil, Ecuador.

### Non tuberculous mycobacteria heat inactivation and DNA isolation

Mycobacterial colonies were harvested from cultures, resuspended in Tris-EDTA (TE) buffer (10 mM Tris–HCl, 1 mM EDTA, pH 8.0), and inactivated at 95°C for 15 min. After heat inactivation, all samples were centrifuged for 5 min at 10,000 g, and the supernatant was directly used for molecular procedures (Aldous et al., 2005).

### Identification of mycobacteria species by matrix-assisted laser desorption/ionization time of flight mass spectrometry (MALDI-ToF MS)

For sample preparation and extraction, a modified version of Bruker Daltonik’s *Mycobacteria* Extraction method version 3 was used (Toney et al., 2022; Orlando et al., 2025). Briefly, to optimize the protein extraction, an incubation at 15 to 25°C was done following the addition of 70% formic acid and again after the addition of acetonitrile. Colonies were taken from a plate and transferred to a tube containing 300 mL of HPLC grade water, then vortexed to get a uniform suspension, and treated at 95°C for 30min. After 5 min of cooling, 900 μL of HPLC grade ethanol was added; the suspension was centrifuged for 2 min at maximum speed, and the supernatant was discarded. Centrifugation was repeated followed by the removal of residual ethanol with a pipette. The pellet was left to air dry for 30 min at room temperature. Then, 50 μL of 70% formic acid was added to the pellet and mixed by pipetting for resuspension and left for 15 minutes at room temperature. Then, a volume equivalent to the size of the re-suspended pellet of 0.5 mm diameter zirconia/silica beads (BioSpec Products, Bartlesville, OK) was added to the suspension. The suspension was lysed using a digital disruptor genie (Scientific Industries, Inc., Bohemia, NY) set at maximum speed for 3 min. Then, 50 μL of acetonitrile was added to the lysate and mixed by pipetting, followed by 5 min of incubation at room temperature. Then, the lysate was placed on the disruptor genie for 2 more min at maximum speed. The lysate was centrifuged for 2 min at maximum speed, and the supernatant was collected. Finally, 1 μL of each supernatant lysate was spotted onto a ground steel target plate (Bruker Daltonik, Bremen, Germany) and air-dried for 5 minutes. Each spot was overlaid with 1 µL of matrix solution (a saturated solution of α-cyano-4-hydroxycinnamic acid in 50% acetonitrile with 2.5% trifluoroacetic acid) and air-dried for another 5 minutes. Spectrum acquisition was carried out on a MALDI-ToF Biotyper Microflex instrument using Flex Control 3.1 software in positive linear mode, with a laser frequency of 60 Hz and a mass range of 2,000 to 20,000 Da. Spectra were accumulated from 240 laser shots per point, and between 20 and 24 high-quality spectra were obtained for each bacterial extract.

Each ground steel target plate included a Bacterial Test Standard (BTS) for instrument calibration. As a positive control, *M. fortuitum* ATCC 6841T was used. Mass spectral analysis was performed using the spectrum view in Flex Analysis software and MALDI-Tof Biotyper 3.1 (Bruker Daltonik GmbH, Bremen, Germany). NTM Identification was done by comparison with main spectrum profiles in the *Mycobacteria* Library version 7.0 (Bruker Daltonics, Bremen,Germany). Species identification was considered positive if the score value exceeded 2.000.

### PCR screening for 16s, hsp65, and rpoB genes and DNA sequencing

Partial gene amplification of *16S*, *hsp65*, and *rpoB* was performed using the primers listed in **Supplementary Table 1**. The PCR reaction for 16S was performed using 7.5 µL of *GoTaq^®^Green Master Mix, 2X* (Promega, Wisconsin, United States) (1X), 0.75 µL for each primer (0.5 µM), 1 µL of NTM DNA, and 5 µL of nuclease-free water for a final volume of 15 µL. For *hsp65* and *rpoB* genes, the PCR reaction contained 7.5 µL *GoTaq^®^Green Master Mix, 2X* (1X), 0.45 µL for each primer (0.3 µM), 1 µL of NTM DNA, and 5.6 µL of nuclease-free water for a final volume of 15 µL. The PCR program for the three partial genes was established as follows: initial denaturation at 96°C for 10 min, 35 cycles at 96°C for 30 s, 63°C for 30 s, 72°C for 30 s, and a final extension step of 72°C for 10min. The amplicons were visualized in a 2% agarose gel in 0.5X Tris-boric acid-EDTA (TBE) buffer at 80 mA for 2 h using the ladder *100 bp Plus Opti-DNA Marker* (Cat. No.: G016, Applied Biological Materials Inc., British Columbia, Canada). The amplicons were sequenced by Sanger sequencing and analyzed with the ABI 3500xL Genetic Analyzer from Applied Biosystems at the Service Department of Universidad de Las Americas (Quito, Ecuador).

### Phylogenetic analysis

Sequences were curated in the *Geneious^®^* v. 11.0.4 program (Biomatters, Geneious, 2024), and then each sequence was identified by identity percentage using the BLASTn algorithm (Altschul et al., 1990). Multiple sequence alignment was performed using ClustalW (Thompson et al., 1994) in MEGA11 (Tamura et al., 2021), having discarded sequences with low quality for alignment. Phylogenetic trees were obtained by using the maximum likelihood method and the Tamura 3-parameter model (Tamura, 1992). The heuristic search’s initial tree was automatically inferred using the Maximum Parsimony method. Gaps in alignment were treated as missing data. The reliability of the tree topology was assessed using 100 bootstrap replicates, and *M. tuberculosis* sequences were used as an outgroup. Sequences obtained in the BLAST results were used as reference sequences. Genetic distances were calculated using the p-distance model, using three decimals to differentiate between sequences. The resulting phylogenetic trees were visualized and annotated using iTOL (Interactive Tree of Life), with color schemes applied to delineate species groups (Letunic and Bork, 2024).

Seven phylogenetic trees were generated: one for each marker considered in this study, and four trees using a multi-locus sequence approach (*16S + rpoB, 16S + hsp65, rpoB + hsp65, and 16S + rpoB + hsp65*). Maximum Likelihood analysis was performed for all trees, using the GTR+G, T92+G, GTR+G, and GTR+G+I models, respectively.

### Statistical analysis

Cohen’s Kappa coefficient was used to assess the level of agreement between the two identification methods, specifically comparing MALDI-ToF MS results with those obtained from each phylogenetic analysis. In cases where at least one result included identification of two possible species (e.g., with 16S or combined hsp65 + rpoB analyses), a weighted version of Cohen’s Kappa was applied to account for partial concordance. All statistical analyses were conducted using Microsoft Excel 365.

## Results

### Positivity rates for NTM identification with 16S, hsp65, and rpoB genes

We obtained results with MALDI-ToF MS on the 59 NTM strains, with acceptable confidence scores in all cases (> 2.000). We identified, with this technique, 29 *Mycobacteroides abscessus*, 19 *Mycolicibacterium fortuitum*, 9 *Mycolicibacterium farcinogenes*, 1 *Mycolicibacterium novocastrense*, and 1 *Mycolicibacterium parafortuitum* (**Table 1**). Regarding Sanger sequencing results, 89.83% (53/59), 79.66% (47/59), and 76.27% (45/59) of NTM strains yielded *16S*, *hsp65*, and *rpoB* sequences, respectively. These sequences generated BLASTn results with identity percentages ranging from 71-100% for 16S, 96.6-100% with *hsp65*, and 84.3-100% for *rpoB*. The results of BLAST analyses are shown in **Supplementary Material 1**.

**Table 1:** Results of identification of nontuberculous mycobacteria with MALDI-ToF MS and the different phylogenetic analysis tests with the independent and combined markers (multilocus sequencing approach).

N° sample	MALDI-ToF MS	Phylogeny - 16S	Phylogeny - rpoB	Phylogeny - hsp65	Phylogeny 16S + rpoB	Phylogeny 16S + hsp65	Phylogeny hsp65 + rpoB	Phylogeny 16S + hsp65 + rpoB
1	*M. abscessus*	*M. abscessus, M. chelonae*	*M. abscessus*	*M. abscessus*	*M. abscessus*	*M. abscessus*	*M. abscessus*	*M. abscessus*
2	*M. abscessus*	*M. abscessus, M. chelonae*	*M. abscessus*	*M. abscessus*	*M. abscessus*	*M. abscessus*	*M. abscessus*	*M. abscessus*
3	*M. abscessus*	*M. abscessus, M. chelonae*	*M. abscessus*	*M. abscessus*	*M. abscessus*	*M. abscessus*	*M. abscessus*	*M. abscessus*
4	*M. abscessus*	*M. abscessus, M. chelonae*	*M. abscessus*	*M. abscessus*	*M. abscessus*	*M. abscessus*	*M. abscessus*	*M. abscessus*
5	*M. abscessus*	*b*	*M. abscessus*	*M. abscessus*	*a*	*a*	*M. abscessus*	*a*
6	*M. abscessus*	*M. abscessus, M. chelonae*	*M. abscessus*	*M. abscessus*	*M. abscessus*	*M. abscessus*	*M. abscessus*	*M. abscessus*
7	*M. abscessus*	*M. wolinskyi, M. jacuzzi*	*M. wolinskyi*	*M. wolinskyi*	*M. wolinskyi*	*M. wolinskyi*	*M. wolinskyi*	*M. wolinskyi*
8	*M. abscessus*	*M. abscessus, M. chelonae*	*M. abscessus*	*M. abscessus*	*M. abscessus*	*M. abscessus*	*M. abscessus*	*M. abscessus*
9	*M. abscessus*	*M. abscessus, M. chelonae*	*M. abscessus*	*M. abscessus*	*M. abscessus*	*M. abscessus*	*M. abscessus*	*M. abscessus*
10	*M. abscessus*	*M. abscessus, M. chelonae*	*M. abscessus*	*M. abscessus*	*M. abscessus*	*M. abscessus*	*M. abscessus*	*M. abscessus*
11	*M. abscessus*	*M. abscessus, M. chelonae*	*M. abscessus*	*M. abscessus*	*M. abscessus*	*M. abscessus*	*M. abscessus*	*M. abscessus*
12	*M. abscessus*	*M. abscessus, M. chelonae*	*M. abscessus*	*M. abscessus*	*M. abscessus*	*M. abscessus*	*M. abscessus*	*M. abscessus*
13	*M. abscessus*	*M. wolinskyi, M. jacuzzi*	*M. wolinskyi*	*M. wolinskyi*	*M. wolinskyi*	*M. wolinskyi*	*M. wolinskyi*	*M. wolinskyi*
14	*M. abscessus*	*M. wolinskyi, M. jacuzzi*	*M. wolinskyi*	*M. wolinskyi*	*M. wolinskyi*	*M. wolinskyi*	*M. wolinskyi*	*M. wolinskyi*
15	*M. abscessus*	*M. abscessus, M. chelonae*	*M. abscessus*	*M. abscessus*	*M. abscessus*	*M. abscessus*	*M. abscessus*	*M. abscessus*
16	*M. abscessus*	*M. abscessus, M. chelonae*	a	*M. abscessus*	*a*	*M. abscessus*	*a*	*a*
17	*M. abscessus*	*M. abscessus, M. chelonae*	*M. abscessus*	*M. abscessus*	*M. abscessus*	*M. abscessus*	*M. abscessus*	*M. abscessus*
18	*M. abscessus*	*M. jacuzzi*	*M. wolinskyi*	*M. wolinskyi*	*M. wolinskyi*	*M. wolinskyi*	*M. wolinskyi*	*M. wolinskyi*
19	*M. abscessus*	*M. wolinskyi, M. jacuzzi*	*M. wolinskyi*	*M. wolinskyi*	*M. wolinskyi*	*M. wolinskyi*	*M. wolinskyi*	*M. wolinskyi*
20	*M. abscessus*	*M. abscessus, M. chelonae*	*M. abscessus*	*M. abscessus*	*M. abscessus*	*M. abscessus*	*M. abscessus*	*M. abscessus*
21	*M. abscessus*	*M. abscessus, M. chelonae*	*M. abscessus*	*M. abscessus*	*M. abscessus*	*M. abscessus*	*M. abscessus*	*M. abscessus*
22	*M. abscessus*	*M. abscessus, M. chelonae*	*M. abscessus*	*M. abscessus*	*M. abscessus*	*M. abscessus*	*M. abscessus*	*M. abscessus*
23	*M. abscessus*	*M. abscessus, M. chelonae*	*a*	*M. abscessus*	*a*	*M. abscessus*	*a*	*a*
N° sample	MALDI-TOF MS	Phylogeny - 16S	Phylogeny - rpoB	Phylogeny - hsp65	Phylogeny 16S + rpoB	Phylogeny 16S + hsp65	Phylogeny hsp65 + rpoB	Phylogeny 16S + hsp65 + rpoB
24	*M. abscessus*	*M. abscessus, M. chelonae*	*a*	*M. abscessus*	*a*	*M. abscessus*	*a*	*a*
25	*M. abscessus*	*M. abscessus, M. chelonae*	*M. abscessus*	*M. abscessus*	*M. abscessus*	*M. abscessus*	*M. abscessus*	*M. abscessus*
26	*M. abscessus*	*M. wolinskyi, M. jacuzzi*	*M. wolinskyi*	*a*	*M. wolinskyi*	*a*	*a*	*a*
27	*M. abscessus*	*M. abscessus, M. chelonae*	*M. abscessus*	*M. abscessus*	*M. abscessus*	*M. abscessus*	*M. abscessus*	*M. abscessus*
28	*M. abscessus*	*M. abscessus, M. chelonae*	*M. abscessus*	*M. abscessus*	*M. abscessus*	*M. abscessus*	*M. abscessus*	*M. abscessus*
29	*M. abscessus*	*M. abscessus, M. chelonae*	*a*	*M. abscessus*	*a*	*M. abscessus*	*a*	*a*
30	*M. farcinogenes*	*M. farcinogenes, M. senegalense*	*a*	*M. houstonense*	*a*	*M. houstonense*	*a*	*M. fortuitum*
31	*M. farcinogenes*	*b*	*a*	*a*	*a*	*a*	*a*	*a*
32	*M. farcinogenes*	*M. farcinogenes, M. senegalense*	*M. farcinogenes*	*M. houstonense*	*M. farcinogenes*	*M. houstonense*	*M. farcinogenes*	*M. farcinogenes*
33	*M. farcinogenes*	*M. farcinogenes, M. senegalense*	*M. farcinogenes*	*M. houstonense*	*M. farcinogenes*	*M. houstonense*	*M. farcinogenes*	*M. farcinogenes*
34	*M. farcinogenes*	*M. farcinogenes, M. senegalense*	*M. farcinogenes*	*M. houstonense*	*M. farcinogenes*	*M. houstonense*	*M. farcinogenes*	*M. farcinogenes*
35	*M. farcinogenes*	*M. farcinogenes, M. senegalense*	*M. farcinogenes*	*M. houstonense*	*M. farcinogenes*	*M. houstonense*	*M. farcinogenes*	*M. farcinogenes*
36	*M. farcinogenes*	*M. farcinogenes, M. senegalense*	*M. farcinogenes*	*M. houstonense*	*M. farcinogenes*	*M. houstonense*	*M. farcinogenes*	*M. farcinogenes*
37	*M. farcinogenes*	*M. farcinogenes, M. senegalense*	*M. farcinogenes*	*M. houstonense*	*M. farcinogenes*	*M. houstonense*	*M. farcinogenes*	*M. farcinogenes*
38	*M. farcinogenes*	*M. farcinogenes, M. senegalense*	*M. farcinogenes*	*M. houstonense*	*M. farcinogenes*	*M. houstonense*	*M. farcinogenes*	*M. farcinogenes*
39	*M. fortuitum*	*b*	*a*	*a*	*a*	*a*	*a*	*a*
40	*M. fortuitum*	*M. abscessus, M. chelonae*	*M. farcinogenes*	*M. houstonense*	*M. farcinogenes*	*M. houstonense*	*M. farcinogenes, M. fortuitum*	*M. farcinogenes*
41	*M. fortuitum*	*b*	*a*	*a*	*a*	*a*	*a*	*a*
42	*M. fortuitum*	*b*	*M. wolinskyi*	*a*	*a*	*a*	*a*	*a*
43	*M. fortuitum*	*M. fortuitum*	*M. fortuitum*	*a*	*M. fortuitum*	*a*	*a*	*a*
44	*M. fortuitum*	*M. abscessus, M. chelonae*	*M. abscessus*	*M. abscessus*	*M. abscessus*	*M. abscessus*	*M. abscessus*	*M. abscessus*
45	*M. fortuitum*	*a*	*a*	*a*	*a*	*a*	*a*	*a*
46	*M. fortuitum*	*a*	*M. wolinskyi*	*a*	*a*	*a*	*a*	*a*
47	M. fortuitum	*b*	*a*	*a*	*a*	*a*	*a*	*a*
48	*M. fortuitum*	*b*	*a*	*a*	*a*	*a*	*a*	*a*
49	*M. fortuitum*	*a*	*a*	*a*	*a*	*a*	*a*	*a*
50	*M. fortuitum*	*a*	*a*	*a*	*a*	*a*	*a*	*a*
51	*M. fortuitum*	*a*	*a*	*M. fortuitum*	*a*	*a*	*a*	*a*
52	*M. novocastrense*	*b*	*M. novocastrense*	*M. malmesburyense*	*a*	*a*	*M. novocastrense*	*a*
53	*M. parafortuitum*	*M. parafortuitum*	*M. parafortuitum*	*M. parafortuitum*	*M. parafortuitum*	*M. parafortuitum*	*M. parafortuitum*	*M. parafortuitum*
54	*M. fortuitum*	*M. fortuitum*	*M. fortuitum*	*M. fortuitum*	*M. fortuitum*	*M. fortuitum*	*M. fortuitum*	*M. fortuitum*
55	*M. fortuitum*	*M. fortuitum*	*M. fortuitum*	*M. fortuitum*	*M. fortuitum*	*M. fortuitum*	*M. fortuitum*	*M. fortuitum*
56	*M. fortuitum*	*M. fortuitum*	*M. fortuitum*	*M. fortuitum*	*M. fortuitum*	*M. fortuitum*	*M. fortuitum*	*M. fortuitum*
57	*M. fortuitum*	*M. fortuitum*	*M. fortuitum*	*M. fortuitum*	*M. fortuitum*	*M. fortuitum*	*M. fortuitum*	*M. fortuitum*
58	*M. fortuitum*	*b*	*M. novocastrense*	*M. novocastrense, M. komanii*	*a*	*a*	*M. novocastrense*	*a*
59	*M. fortuitum*	*M. fortuitum*	*M. fortuitum*	*M. fortuitum*	*M. fortuitum*	*M. fortuitum*	*M. fortuitum*	*M. fortuitum*

a: no PCR amplification; b: poor quality sequence (each color is associated to an specific NTM specie; grey color is associated with no identification of NTM species).

### Phylogenetic analysis of 16S, hsp65, and rpoB single sequences for NTM species identification and comparison with MALDI-ToF MS

**Figure 1:**
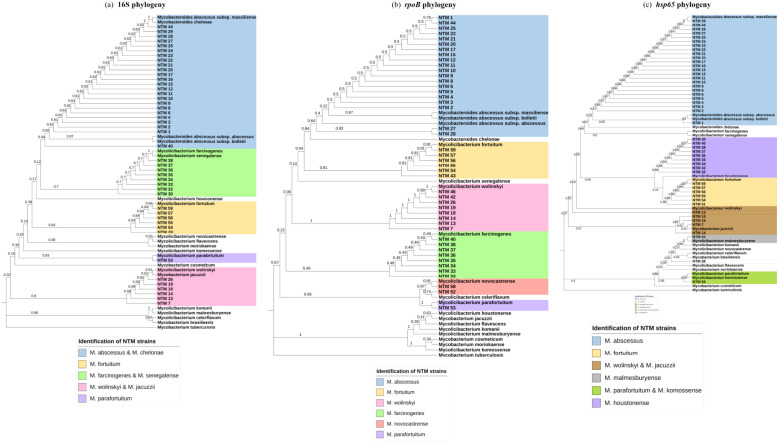
Phylogenetic analysis for individual markers. **(a)** Maximum Likelihood tree (T92+G+I) 16S partial gene 587 nucleotides. **(b)** Maximum Likelihood tree (T92 +G+I) rpoB partial gene 357 nucleotides. **(c)** Maximum Likelihood tree (T92+G) hsp65 partial gene 393 nucleotides.

The results of MALDI-ToF MS and phylogenetic analyses of *16S, hsp65, and rpoB* single sequences are shown in **Table 1** and **Figure 1**. For *16S rRNA*, a total of 45 sequences from 59 clinical isolates were analyzed and compared with MALDI-ToF MS results; only eight strains could be identified at the species level, and thirty of them were partially concordant with the MALDI-ToF MS technique; the weighted Cohen’s Kappa value was 0.46. For *hsp65*, a total of 47 sequences from the 59 clinical isolates were analyzed and compared with MALDI-ToF MS results; 46 could be identified to the species level but with a weighted Cohen’s Kappa value of 0.51; *M. farcinogenes* and *M. novocastrense* could not be identified with this marker. For *rpoB*, a total of 45 sequences from 59 clinical isolates were analyzed and compared with MALDI-ToF MS results; 34 could be identified at the species level, weighted Cohen’s Kappa value was 0.69.

### Phylogenetic analysis of concatenated 16S, hsp65, and rpoB sequences for NTM species identification and comparison with MALDI-ToF MS

**Figure 2:**
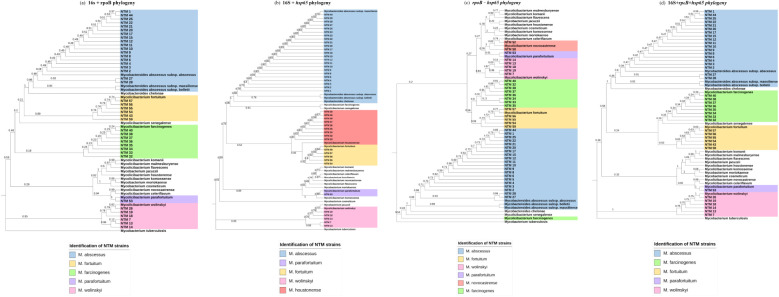
Phylogenetic analysis for concatenated markers. **(a)** Maximum Likelihood tree (T92+G) 16S + *rpoB* partial gene 702 nucleotides. **(b)** Maximum Likelihood tree (T92+G) 16S + *hsp65* partial gene 1002 nucleotides. **(c)** Maximum Likelihood tree (T92+G) *rpoB* + *hsp65* partial gene 1620 nucleotides. **(d)** Maximum Likelihood tree (GTR+G+I) 16S+*rpoB*+*hsp65* partial genes 1081 nucleotides.

The results of MALDI-ToF MS and phylogenetic analyses of concatenated *16S, hsp65, and rpoB* sequences are shown in **Table 1** and **Figure 2**. For *16S + hsp6* genes, a total of 43 concatenated sequences were considered in comparison with MALDI-ToF MS results (strains with only one sequence between the two markers were discarded); in all 43, identification to species level was achieved, although without identifying *M. farcinogenes* and *M. novocastrense*; cohen’s Kappa value was 0.71. For *16S + rpoB* genes, a total of 40 concatenated sequences were considered for comparison with MALDI-ToF MS results (strains with only one sequence between the two markers were discarded); in all 40, identification to species level was achieved, although without identifying *M. novocastrense*; Cohen’s Kappa value was 0.76.

For *hsp65 + rpoB* genes, a total of 41 concatenated sequences were considered for comparison with MALDI-TOF MS results (strains with only one sequence between the two markers were discarded); in all 41, identification to species level was achieved, although without identifying *M. novocastrense*; Cohen’s Kappa value was 0.69.

Finally, concatenated analysis for the three genes *(16S + hsp65 + rpoB)* was also done (**Figure 2D**). A total of 38 concatenated sequences were considered for comparison with MALDI-ToF MS results (strains with only one or two sequences between the three markers were discarded). In all 38, identification to species level was achieved, although without identifying *M. novocastrense*. Cohen’s Kappa value was 0.72. For a summarized view of Cohen’s Kappa results for the different phylogenetic analyses, see **Table 2**.

**Table 2:** Cohen’s Kappa results for the concordance measurements between the techniques used in this study.

MALDI-ToF MS vs phylogeny of ...	Cohen’s Kappa result
16S	0.46
hsp65	0.51
rpoB	0.69
16S + hsp65	0.71
16S + rpoB	0.76
hsp65 + rpoB	0.69
16S + hsp65 + rpoB	0.72

## Discussion

The global rise in NTM infections is a growing public health concern, particularly due to the associated challenges in diagnosis and treatment (Zhang et al., 2023). Despite this alarming trend, there is a notable scarcity of comprehensive epidemiological studies on NTM (Dohál et al., 2023), especially in LMICs where healthcare systems often face significant resource constraints affecting diagnosis. This highlights an urgent need to develop and evaluate diagnostic tools that are not only accurate but also cost-effective, ensuring their accessibility in regions where financial and technical limitations may hinder advanced diagnostic approaches.

On the other hand, it is crucial to emphasize the importance of conducting validation studies for clinical diagnosis tools in LMICs, utilizing biological samples derived from these regions. The high diversity of mycobacterial species and diverse geographical distribution patterns of species or strains, coupled with the limited characterization of many of them (Zhang et al., 2023) could raise the possibility of discrepancies in diagnostic outcomes when using traditional molecular markers in different epidemiological scenarios. Local studies are essential to uncover region-specific variations, refine diagnostic tools, and enhance the reliability of identification methods in diverse epidemiological settings (Garcia-Bereguiain et al., 2022; Morales-Jadan et al., 2023). In this sense, to the best of our knowledge, this is the first study of its kind not only in Ecuador but also in South America.

It is well known that NTM are difficult to identify by genetic methods due to their complex taxonomy. In this sense, efficient, rapid, and accurate methods for species identification are of great importance. MALDI-ToF MS is considered a gold standard for the identification of NTM species in human clinical practice (Schubert and Kostrzewa, 2017; Alcolea-Medina et al., 2019; Costa-Alcalde et al., 2019; Lorente-Leal et al., 2022; Rodriguez-Temporal et al., 2022; Toney et al., 2022; Zhu et al., 2024). However, MALDI-ToF MS availability is limited in LMICs. Moreover, while different protocols for NTM identification are available based on Sanger sequencing of different gene targets, the information regarding their performance is still scarce (Lorente-Leal et al., 2022). For instance, a previous report showed that MALDI-ToF MS results with a score above 1.85 are equivalent to partial sequencing of the *rpoB* gene (Costa-Alcalde et al., 2019). In general, the studies available support that MALDI-ToF MS is an effective method for rapid screening of NTM and detection of new NTM species (Schubert and Kostrzewa, 2017; Alcolea-Medina et al., 2019; Costa-Alcalde et al., 2019; Lorente-Leal et al., 2022; Rodriguez-Temporal et al., 2022; Toney et al., 2022; Zhu et al., 2024), while Sanger sequencing could be implemented as an additional method when MALDI-ToF MS is not available or for further characterization of NTM species (Costa-Alcalde et al., 2019; Kim et al., 2022; Lorente-Leal et al., 2022). More specifically, the Bruker Biotyper MALDI-ToF MS platform has also been evaluated with an extensive number of NTM isolates previously identified by Sanger DNA sequencing of the full-length *16S rRNA* and *rpoB* gene (Alcolea-Medina et al., 2019), showing an accuracy of 94%. Interestingly, while MALDI-ToF MS performed well against Sanger sequencing of the *16S rRNA* gene alone, some NTM species required additional sequencing of *rpoB*. Moreover, most discrepancies between MALDI-ToF MS and sequencing results are usually due to underrepresentation of some species in the libraries used, and it is expected to improve with updated libraries (Toney et al., 2022). Considering these references, we took advantage of the only MALDI-ToF MS (Bruker Biotyper MALDI-TOF MS platform) laboratory for bacterial identification available in Ecuador, to use NTM identification with this method as a gold standard to compare with Sanger sequencing-based methods available at several locations within the country.

The genes 16S rRNA, *hsp65*, or *rpoB* have been traditionally used as a reference standard for identifying NTM (Huang et al., 2020). This approach involves the amplification, sequencing, BLAST-n, and phylogenetic analysis of one or more conserved genes. Consistent with previous studies, our findings highlight the limitations of the 16S marker in distinguishing between closely related NTM species, especially within the rapidly growing mycobacteria groups (Tortoli, 2010; Maleki et al., 2017). Some NTM species may have ambiguous bases within this gene, potentially due to the presence of multiple non-identical copies of the gene, making identification difficult (Takeda et al., 2018).

In our study, this marker allowed species-level identification for only 8 out of 45 sequences, while 30 out of 45 results showed partial concordance with MALDI-ToF MS. For instance, *M. abscessus* and *M. chelonae* could not be differentiated using 16S, as the gene exhibits high similarity between these species (Zou et al., 2014). On the other hand, phylogenetic analyses with *hsp65* and *rpoB* did generate species-level identifications for almost all strains that could be sequenced with these markers, which agrees with other studies (Adékambi et al., 2003; Lee et al., 2003; Maleki et al., 2017; Kim and Shin, 2018).

However, *hsp65* was not able to identify *M. farcinogenes* and *M. novocastrense*. Eight strains identified as *M. farcinogenes* by MALDI-ToF MS (strains 30, 32–38) were classified as *M. houstonense* based on *hsp65* sequence analysis, but the *16S rRNA*, *rpoB*, and three out of four concatenated analyses supported the MALDI-ToF MS identification as *M. farcinogenes*. Similarly, strain 52 was identified as *M. novocastrense* by MALDI-ToF MS, while *hsp65* analysis suggested *M. malmesburyense*, but the *rpoB* marker and the concatenated analysis were consistent with the MALDI-ToF MS result. These findings indicate that the *hsp65* marker alone may not provide sufficient resolution to distinguish between *M. farcinogenes* and *M. houstonense*, or between *M. novocastrense* and *M. malmesburyense*. Although this gene is widely used for NTM species identification because of its variability compared to other markers such as 16S rRNA, there is no standard pattern for all species; therefore, the combination of two or more markers (MLST approach) is necessary to resolve identification discrepancies (Moghim et al., 2012; Kim and Shin, 2018).

A previous study reported NTM species identification rates of 71.3%, 86.79%, and 81.55% using the 16S rRNA, *hsp65*, and *rpoB* genes, respectively. Notably, the identification rate increased to 97.25% when a multi-locus sequence analysis (MLSA) combining all three markers was applied (Rodriguez-Temporal et al., 2022). These findings align with our results, which demonstrate improved accuracy and discriminatory power for NTM species identification through concatenated analyses of multiple gene sequences, enhancing both resolution and the robustness of phylogenetic trees (Devulder et al., 2005). In our MLSA, species-level concordance values increased significantly: 0.71 for 16S + *hsp65*, 0.76 for 16S + *rpoB*, 0.69 for *hsp65* + *rpoB*, and 0.72 for the concatenated analysis of all three genes. These results underscore that any of the multi-locus approaches provide greater species-level resolution than analyses based on a single gene alone.

It is noteworthy that phylogenetic analysis using the *rpoB* marker and the four concatenated trees enabled subspecies-level identification of 18 isolates as *Mycobacterium abscessus* subsp. *abscessus* (strains 1–6, 8–11, 15, 17, 20–22, 25, and 44). A few reports have also been able to demonstrate the utility of Sanger sequencing to discriminate subspecies within the *M. abscessus* complex (Simmon et al., 2007; Nie et al., 2014). However, this level of resolution was not included in the comparative analysis between methods, as MALDI-ToF MS provided identifications only at the species level. MALDI-ToF MS generally lacks the resolution to distinguish NTM subspecies, with its accuracy largely dependent on the availability and quality of reference spectra (Schubert and Kostrzewa, 2017; Lorente-Leal et al., 2022; Rodriguez-Temporal et al., 2022; Calderaro and Chezzi, 2024; Zhu et al., 2024). As such, the comparative analysis in this study was intentionally limited to species-level identifications to align with the validated capabilities of MALDI-ToF MS and to ensure consistency across methods. Subspecies-level identification often holds important clinical implications due to the distinct antimicrobial resistance profiles of its members. For instance, *M. abscessus* subsp. *abscessus* and subsp. *bolletii* could exhibit inducible resistance to macrolides, mediated by a functional *erm(41)* gene. In contrast, *M. abscessus* subsp. *massiliense* carries a truncated, non-functional version of *erm(41)* and is typically more susceptible to macrolide-based therapies (Lee et al., 2015; Bao et al., 2022). Accurate subspecies differentiation is therefore crucial for guiding appropriate antimicrobial treatment, and this capability could be an advantage of using Sanger sequencing compared to MALDI-ToF MS.

In conclusion, our study carried out with an extensive collection of NTM isolates from Ecuador points out that a multi-locus approach for Sanger sequencing, especially combining *16S + rpoB* genes, is an alternative, accurate method for NTM species identification in the absence of MALDI-ToF MS. These findings should be considered by the regional public health authorities to provide guidelines to laboratories using Sanger sequencing for NTM species identification within Ecuador.

## Data Availability

The original contributions presented in the study are included in the article/**Supplementary Material**. Further inquiries can be directed to the corresponding author.
