# Supplementary material for: Comparative evaluation of MALDI-ToF mass spectrometry and Sanger sequencing of the 16S, hsp65, and rpoB genes for non tuberculous mycobacteria species identification
Source: Front Cell Infect Microbiol. 2025 Jul 28;15:1612459. doi: 10.3389/fcimb.2025.1612459 (PMC12336121; doi:10.3389/fcimb.2025.1612459)
Supplement: Supplementary file 2 [file Table1.docx]

**Supplementary Table 1. Primers used for PCR reactions and in Sanger sequencing.**

| **Gene** | **Name** | **Sequence 5'-3'** | **Amplicon size (bp)** |
| --- | --- | --- | --- |
| 16S [38] | A1F | CTGGCTCAGGACGAACGCTG | 600-630 |
|  | 54R | TCTAGTCTGCCCGTATCGCCC |  |
| *rpoB* [37] | RPO5’ | TCAAGGAGAAGCGCTACGA | 360 |
|  | RPO3’ | GGATGTTGATCAGGGTCTGC |  |
| *hsp65* [14] | Tb11 | ACCAACGATGGTGTGTCCAT | 439 |
|  | Tb12 | CTTGTCGAACCGCATACCCT |  |
